# High and highly bonded: Fused football fans who use cocaine are most likely to be aggressive toward rivals

**DOI:** 10.1016/j.drugpo.2021.103263

**Published:** 2021-07

**Authors:** Martha Newson

**Affiliations:** a School of Anthropology and Conservation, University of Kent, Canterbury, Kent, UK; b Centre for the Study of Social Cohesion, University of Oxford, Oxford, UK

**Keywords:** Cocaine, Identity fusion, Football fans, Aggression, Social bonding, Intergroup violence

## Abstract

**Background:**

Cocaine use among British football fans has yet to be quantified, despite its association with football-related aggression by the media. Identity fusion, a potent form of social bonding, is a well-established predictor of fan violence, which has never been investigated in relation to stimulant use.

**Methods:**

British football fans (*n* = 1486) completed a self-selected online survey about how bonded they were to their club and fellow fans, their cocaine use, and aggressive behavior toward rival fans over the prior 12-months.

**Results:**

Fans reported more cocaine use than the national average (6.19%). Of fans who attended stadia matches, 1.08% had used cocaine in stadia, and 30.05% had witnessed other fans take cocaine at matches. Fans who used cocaine reported significantly more aggression toward rivals than fans who did not. Cocaine and identity fusion significantly interacted, such that highly ‘fused’ cocaine users were especially likely to have behaved aggressively toward their rivals. Men were more fused and more aggressive than women.

**Conclusion:**

Football mirrors wider society, be it in social issues or drug use. The widespread availability of cheap but high purity cocaine may be associated with fan aggression, especially among fans who are highly bonded to their clubs and fellow fans.

## Highlights

• A sample of 1486 British football fans completed an online survey.
• More fans reported using cocaine than the national average.
• Around 1% of fans reported cocaine use in stadia, but 30% had seen others use it.
• Identity fusion, an intense form of bonding, predicted aggression toward rivals.
• Cocaine use and identity fusion significantly interacted to predict aggression.

## Introduction

Cocaine is the second most prevalent classified drug in the UK (ONS, 2020). Its stimulant properties lead to intense bursts of euphoria, strength and confidence, whilst simultaneously supressing inhibitions, fatigue, appetite, and pain ACMD (2015). This could make it highly desirable to individuals who find themselves on the ‘frontline’ during intergroup conflicts. For instance, cocaine is often the drug of choice in football ‘firms’ (Ayres & Treadwell, 2012). However, these potential gains come at a cost. Beyond executive function deficits, chronic use is also associated with socio-cognitive impairments, including reduced empathy and prosocial behavior, and increased Machiavellianism (Quednow, 2016).

Perhaps unsurprisingly, then, cocaine is a strong predictive factor for violence in drug treatment centres (Chermack & Blow, 2002), though it is also possible that a predilection for violence is also predictive of taking cocaine. Cocaine's relationship with alcohol is complex, both pharmacologically and culturally. The potential interactive effect of alcohol and cocaine, through the production in the liver of highly toxic cocaethylene, may magnify the effects of both drugs, including increased violent thoughts and behaviours (Pennings, Leccese, & Wolff, 2002). Nonetheless, among young people in the UK, cocaine has been established as a predictive factor for violence, independently from alcohol (Lightowlers & Sumnall, 2014).

Football fans may be a population where the aggressive outcomes associated with cocaine use are amplified. Cocaine use among football fans has already been associated with the construction of ‘(hyper-) masculine identities’ and associated aggression (Ayres & Treadwell, 2012). Indeed, cocaine has become an element of ‘Lad’ culture and, alongside alcohol, fuels competitiveness and aggression from travel to a match, until well after it is finished. Similarly, the masculinities embedded in ‘hooligan’ football cultures continue to reverberate through the generations, which normalize violence, especially in the night time economy (Ellis, 2015). Importantly, how female football fans navigate this masculinised world of violent football fandom is not well researched (Pope, 2013). Just as with alcohol, cocaine may be too heavily blamed for fan aggression. For instance, there are large fan cultures who drink heavily, but are known for their peaceful fan interactions, e.g., Irish national team fans and fans of Scandinavian clubs (Pearson & Stott, 2016).

Cocaine has not yet been targeted by specific interventions within football, yet calls for inquiries into its use have been made by the British police and have been the subject of media discourse. Despite a doubling in cocaine-related arrests making headline news in 2019, actual figures are low – just 83 police reports of fan cocaine use or possession in 2019/20 (Home Office, 2020). This paper first addresses the subject of cocaine use among fans by surveying them in a confidential survey about their football-based cocaine use.

The second question this paper addresses is whether self-reported cocaine use interacts with fan bonding to predict aggressive behaviours toward rival fans and what gender differences might exist. A well-established predictive factor of inter-group violence is identity fusion, an extreme bonding to one's group, whereby one's sense of self becomes totally immersed in one's group identity (Swann, Jetten, Gómez, Whitehouse, & Bastian, 2012; Whitehouse, 2018). When ‘fused’, individuals feel emboldened to engage in extreme pro-group behaviours due to the sense that their group is personally invested in them. This relationship between fusion to one's group and fighting, or even laying down one's life for the group, is found in high-conflict groups around the world, e.g., militia and terrorist cells, as well as in football fans (Newson et al., 2018; Newson, Buhrmester, & Whitehouse, 2021; Whitehouse, 2018).

To date, fusion has not yet been studied in conjunction with drug taking, but it may offer a fresh approach hinging around individual-level differences, extending crowd psychology arguments around football violence (Pearson & Stott, 2016). While ecstasy and the ‘group love’ it resulted in arguably contributed to the fall in British ‘hooliganism’ in the 1990s (Taylor & Taylor, 1997), does cocaine use help sustain the darker, potentially aggressive side of football fandom?

## Method

In May 2020, 1500 participants completed an online survey via Prolific. Only participants who watched football, lived in the UK, and were over 18 were invited. Participants were reimbursed an equivalent of £7.52 p/h. Two manipulation checks were included, both of which were failed by 13 participants who were excluded, and one participant had incomplete data leaving *n* = 1486 (*M* age = 38.66, *SD* = 13.16, 75% male). Six people chose not to report their gender and were not included in gender analyses. Ethical approval was granted from the University of Oxford (SAME_C1A_20_005).

### Measures

First, participants stated what league their club was in; their age; and how often they had watched football or attended stadia matches over the previous 12-months (*less than once a year, once or twice a year, monthly, weekly, daily*). Next, they answered the verbal identity fusion scale, with reference to their fellow fans (Gómez et al., 2011, Cronbach's α = 0.94). Participants then reported whether they had ever used cocaine. If they answered ‘yes’, they were asked if they had used it within the last 12-months and, if they had, how frequently they had taken it while watching football, in stadia, or outside of football (*never, once or twice, monthly, weekly, daily*). All participants who had attended at least one stadium match in the last 12-months were asked whether they had seen others use cocaine at matches (*never, once or twice, several times, regularly, all the time*). Finally, aggressive behavior was measured with fans’ self-reports of behavior toward rival fans over the last 12-months, including *swearing at them, insulting them, shouting at them, spitting at them, throwing drinks or objects at them*, and *punching, kicking or otherwise assaulting them*. Participants could respond never (0), sometimes (1), or often (2), from which a total aggression score was computed (Cronbach's α = 0.78). As this data was collected at the start of the pandemic, participants were asked to think about their behavior ‘before the pandemic, in the last 12-months’. Rates may therefore be somewhat lower than other 12-month periods, due to football match restrictions in the UK.

### Statistical analysis

Men and women's cocaine use over the last 12 months (no = 1, yes = 2) and aggressive behaviours were statistically compared using chi-squared and Mann Whitney-U tests respectively. Two linear regressions were conducted with aggression the dependent variable. In the first, fusion, age, and gender (men = 1, women = 2) were entered as predictors. In the second, cocaine use and its interaction with fusion were also added as predictors. Identity fusion was treated as a continuous variable and its mean score used for analysis as consistent with past research using the verbal scale (Gómez et al., 2011). Aggression was also treated as a continuous variable, but it had severe negative kurtosis due to a near floor response (kurtosis = 4.16, *SE* = 0.16). Mann Whitney-U and OLS were determined appropriate for this non-normal data. All data analyses were conducted using JASP (v0.12.2). The dataset is available via OSF: https://osf.io/6k7vh/files/.

## Results

Participants supported clubs in the Premiership (78.24%), Championship (13.63%), and Leagues 1 and 2 (8.14%). Overall, 6.19% of participants had used cocaine in the previous 12-months. The sample had an average age of 38.66, yet the results revealed higher cocaine use than that reported by the ONS (2.9%), more closely aligned to cocaine use among 16–24 year olds (6.2%). Of participants who reported cocaine use in the last 12-months, 35.87% had used cocaine at least once while watching football, and 17.39% had used it at a stadium (2.22% and 1.08% of the total sample respectively), see Table 1. In addition, 30.05% of participants who had visited stadia in the last year (*n* = 863) reported witnessing other fans using cocaine at football matches. Men (7.08%) and women (3.57%) were equally as likely to have used cocaine in the previous 12-months (*p* = .681); the sample became too small for further meaningful analyses as only one woman reported using cocaine in stadia (0.28%), compared to 1.34% of men (*p* = .587).

**Table 1:** Descriptive statistics of cocaine use and football-related aggression.

Frequency of use	Fans who reported using cocaine when watching football %	Fans who reported using cocaine in stadia* %	Fans who reported witnessing others use cocaine at matches* %
*Never*	3.97	5.11	69.95
*Once or twice*	1.55	0.94	17.40
*Several times*	0.47	0	8.12
*Regularly*	0.20	0.14	3.60
*all the time*	0	0	0.93

Behavior to rival fans in the last 12 months⁎⁎	Fans who used cocaine				Fans who did not use cocaine		
*Swore at them*	*N*	*M*	*SD*		*N*	*M*	*SD*
*Insulted them*	133	0.50	0.66		65	0.85	0.80
*Shouted at them*	77	0.73	0.70		38	0.90	0.80
*Spat at them*	77	0.68	0.68		38	1.03	0.75
*Thrown drinks or other objects at them*	133	0.02	0.15		65	0.03	0.25
*Punched, kicked or otherwise assaulted them*	133	0.02	0.15		65	0.08	0.32

⁎ Those who visited a stadium in the last 12 months.

⁎⁎ (0 = never, 1 = sometimes, 2 = often).

Most participants reported very little aggression toward rivals in the last 12-months, creating a near floor effect (*M* = 1.20, *SD* = 1.70, range = 0–12). Fans who had used cocaine were significantly more likely to report aggression toward rivals (*M* = 2.14, *SD* = 2.52) than fans who had not (*M* = 1.38, *SD* = 1.80), *t*(196) = −2.44, *p* = .016, Cohen's *d* = 0.37. This was true for all aggression items, but after Bonferroni-corrected t-tests, only significantly so for swearing at rivals *t*(196, = −3.28, *p* = .001, Cohen's *d’s* = 0.50). Men were significantly more likely to report aggression (*M* = 1.36, *SD* 1.77) than women (*M* = 0.17, *SD* = 1.77), *U* = 72,810.5, *p* < .001, Rank-Biserial = 44.

Identity fusion (*M* = 3.59, *SD* = 1.47, range = 1–7) was higher in men (*M* = 3.66, *SD* 1.47), compared to women (*M* = 3.38, *SD* = 1.46), *U* = 205,625, *p* = .003, Rank-Biserial = 11. Highly fused people (*β* = 0.25, *p* < .001), younger people (*β* = −0.12, *p* < .001), and men (*β* = −0.22, *p* < .001) were all significantly more likely to report aggressive behaviours, *R*^2^ = 0.13, *F*(3, 917) = 47.19, *p* < .001. In a second model, including cocaine and its interaction with fusion, there was only a significant main effect of gender (*β* = −0.22, *p* < .001; other main effects, *p* > .070), but there was a 2-way interaction between fusion and cocaine (*β* = 0.75, *p* = .011), such that highly fused cocaine users were especially likely to report aggressive behaviours, *R^2^* = 0.15, *F*(5, 192) = 7.78, *p* < .001.

## Conclusions

Cocaine use among British football fans was found to be slightly higher than the UK average (6.19%), but relatively low within stadia (1.08%). Nearly a third of stadium-going fans had witnessed others taking cocaine at football matches. Cocaine use was significantly associated with swearing and shouting at rivals. There were positive but non-significant trends for increased aggression at the higher levels of spitting and physical violence, possibly owing to near floor-effects, whereby very few participants reported any football violence at all. This in turn, may in part be due to respondents’ self-selection, but also supports research demonstrating peaceful and co-operative attitudes among many fans and fan groups (e.g., Pearson & Stott, 2016).

In support of previous research (Newson et al., 2018), identity fusion was a predictive factor of past, self-reported inter-group fan aggression. However, when adding cocaine and its 2-way interaction with fusion to the model, it became clear that fusion and cocaine use interacted, such that highly fused individuals who took cocaine were particularly likely to report past aggression toward rivals. This finding is promising in at least two main directions. First, theoretically, drug use has not been studied in conjunction with identity fusion before. Cocaine and other stimulants may embolden individuals via enhanced agency or reduced inhibitions, which in turn amplifies the sense of reciprocal strength felt by highly-fused, bonded individuals.

Second, the cognition underlying fan bonding, specifically identity fusion, has been demonstrated in a number of other, high-risk groups so these findings have the potential to inform a wide reach of researchers and policymakers (Whitehouse, 2018). Thus, this study can help direct future, policy-directed research into drug use by individuals at risk of high-threat intergroup scenarios, e.g., gang members, those living in conflict states, or indeed football fans. Fan bonding relates to a host of extreme behaviours both in the UK (Newson et al., 2021) and beyond (e.g., Brazil, Newson et al., 2018) giving the present findings international reach. Finally, adding to an under-developed literature, men reported significantly more fan aggression and higher fusion than did women, but there were no differences in cocaine use, though the sample size was limited for women (Pope, 2013).

This study has a number of limitations. First, its self-reported design may result in under-reporting of cocaine use and aggression. In particular, participants were self-selecting via their engagement with the Prolific platform and may have been concerned about their reputations, despite the anonymity of the survey. This would explain the discrepancy between observed and self-reported cocaine use at football matches. Online fan studies are unlikely to be representative of the diverse make up of real-world fans. However, this data can be used to triangulate evidence gleaned from observations and interview data (e.g., Ayres & Treadwell, 2012) or police arrests.

Secondly, a causal link between cocaine, identity fusion, and football violence cannot be claimed in this correlational study. Importantly, this study does not unpick the concurrent effects of alcohol on aggression, which many of these fans would have also consumed. Furthermore, the results may point to a link between a substance using lifestyle in general and aggression at football matches (particularly among highly bonded fans), either instead of or in addition to, a specific link between cocaine use and aggression. To better understand the causal relationship, future research could identify samples that are willing to regularly report their drug use and aggressive behavior at live games.

In conclusion, cocaine use among football fans may be higher than the national average, and that even within a relatively low-aggression sample, cocaine use predicted aggression, especially among highly fused people. This study raises questions about how stimulant use in groups prone to conflict ought to be best managed. Finally, the findings may support further research into the interplay between drug use, social identity psychology, and policy development.

## Funding

This work was funded by a UKRI Future leaders Fellowship grant (MR/T041099/1).

## Ethics

The sample completed an online survey, including sensitive and personal data. The survey was anonymous. Ethical approval was granted.

## Declarations of Interest

None.
